# Utility of the I-index as a pre-screening tool for total bilirubin testing: a retrospective study in a regional hospital

**DOI:** 10.1515/almed-2025-0161

**Published:** 2026-01-22

**Authors:** Amanda Jiménez García, Manuel Ruiz Artero, Carmen Garcia Rabaneda, Elena Pizarro Peña, Félix Gascón Luna

**Affiliations:** 221796 Hospital Valle De Los Pedroches, Análisis Clínicos, Pozoblanco, Cordoba, Spain; Análisis Clínicos Intercentros, Hospital Universitario Campus de La Salud, Granada, Spain; Hospital Valle De Los Pedroches, Pozoblanco, Andalucía, Spain; Análisis Clínicos, Hospital Valle De Los Pedroches, Pozoblanco, Córdoba, Spain

**Keywords:** total bilirubin, diagnostic performance, hyperbilirubinemia, icteric index

## Abstract

**Objectives:**

This study evaluates the efficacy of the icteric index as a pre-screening tool for hyperbilirubinemia in adult patients.

**Methods:**

The use of this index would help optimize the use of clinical laboratory resources.

**Results:**

A retrospective analysis of 3,194 serum samples revealed a strong correlation between icteric index and total bilirubin levels (r=0.972), with an area under the ROC curve of 0.90. An index cut-off value of 1.2 yielded a sensitivity of 100 % and a specificity of 87 %. This performance guarantees the detection of the totality of hyperbilirubinemia cases and the absence of false negatives, albeit with some false positives. This method would avoid around 79 % of unnecessary total bilirubin tests, resulting in estimated annual cost savings of over 1,500 Euros.

**Conclusions:**

In conclusion, the icteric index is an effective, inexpensive, and safe tool for improving the diagnostic performance of laboratory tests without compromising the quality of care.

## Introduction

The key role of the clinical laboratory is not processing samples, but providing safe and reliable data that support diagnosis and therapeutic decision-making. Certainly, the majority of medical decisions are based on laboratory results. However, this reliance has brought about a predictable effect: the steadily rising demand for diagnostic tests [1].

This often uncontrolled increase does not always translate into a real clinical benefit. Ordering unnecessary tests may lead to erroneous results – primarily false positives – that may lead to additional inappropriate testing. The laboratory faces the challenge of preserving its utility without becoming a source of unnecessary costs.

A good example of this situation is total bilirubin test usage. Total bilirubin (TBil) is requested in routine clinical practice, often without a clear clinical indication. Manifestations of elevated bilirubin levels, known as hyperbilirubinemia, include jaundice, and a yellowish pigmentation of the skin and the sclera caused by bilirubin build-up. Very high concentrations of bilirubin (425 micromol/L) cause toxicity from unconjugated bilirubin, which diffuses across membrane lipids resulting in cell function deterioration, especially in the nervous system.

Based on the origin of hyperbilirubinemia, jaundice is classified as prehepatic, hepatic, and posthepatic.

Icteric index (I-index) is a practical *in vitro* test used for the semi-quantitative determination of bilirubin levels in serum or plasma. This low-cost tool may be used for pre-screening or as part of a clinical algorithm to determine when bilirubin quantification is really necessary, thereby avoiding inappropriate testing [2].

The objective of this study was to assess the efficacy of the I-index as a preliminary screening marker to identify patients with total bilirubin values exceeding the upper limit of normality for the healthy adult population. Serum indices are routinely included in all clinical biochemistry test orders.

## Materials and methods

We carried out a retrospective study of TBil levels and their corresponding I-index values for routine and emergency tests performed in our hospital in January 2025. The two parameters were quantified on an Atellica Solution (Siemens Helathineers; Germany) autoanalyzer.

I-index was determined by calculating absorbance measurements in samples diluted with 0.9 % sodium chloride at 480 nm (primary wavelength) and 505 nm (secondary wavelength).

TBil was measured using the Atellica CH Total Bilirubin assay, which is based on a chemical oxidation method employing vanadate as the oxidizing agent.

A logistic regression and correlation study were performed to assess the correlation between I-index and TBil. Additionally, we assessed the diagnostic performance of the I-index by ROC curve analysis taking TBil values as a reference to differentiate normal (<1.2 mg/dL) from abnormal values. Metrics included sensitivity, specificity, positive predictive value and negative predictive value.

All statistical analyses were performed using the Medcalc Statisticalc Software package (version 23.2; Medcalc Software Ltd, Ostende, Belgium).

## Results

A total of 3,194 urgent and routine serum TBil tests were performed during the study period. Orders from the Unit of Neonatology (18 TBil requests) were also included. The I-index was calculated for all samples. A total of 8.67 % of samples indicated hyperbilirubinemia [3].

Hyperbilirubinemia was defined as a total bilirubin value>1.2 mg/dL. A value below that limit was considered normal and patients were considered healthy.

**Figure 1:**
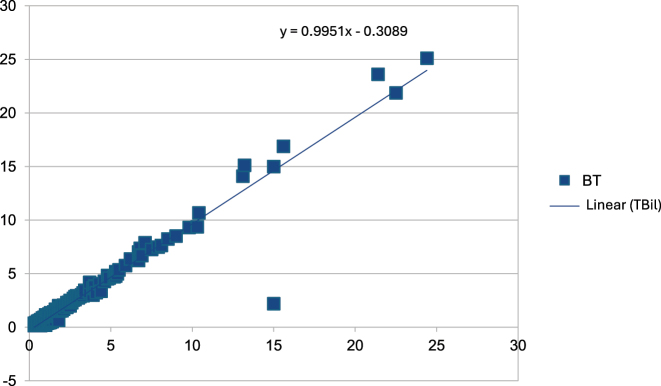
Total bilirubin regression equation.

A correlation study was carried out to assess the relationship between TBil and I-index. Pearson correlation coefficient (r) was 0.972. The regression equation was TBil= (0.9951*I-index)-0.3089 (Figure 1).

**Figure 2:**
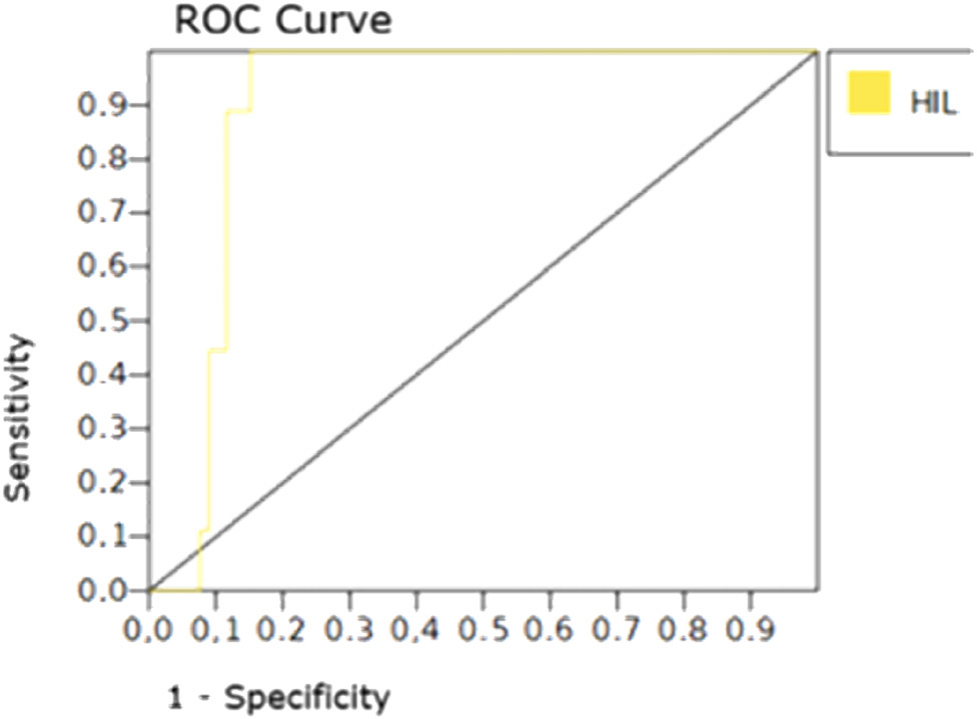
ROC curve for the icteric index at a cut-off value of 1.2 mg/dL.

The area under the ROC curve (AUC) was 0.90 (95 %CI 0.89–0.92 %) for a cut-off value of 1.2 mg/dL (Figure 2). Sensitivity was 100 % (95%CI 98.7–100) and specificity 87 % (95 %CI 85.7–88.14 %), with a positive predictive value of 42 % and a negative predictive value of 100 % (Table 1).

**Table 1:** Sensitivity, specificity, positive predictive value and negative predictive value.

AUC	Sensitivity	Specificity	Positive predictive value	Negative predictive value
0.90 95 %CI (0.89–0.92 %)	(100 %) (95 %CI) (98.7–100)	(87 %) (95 %CI)(85.7–88.14 %)	42 %	100 %

(95 %CI, 95 %) confidence interval.

## Discussion

Based on the results of our study, when the total bilirubin test is included in a biochemistry order, a TBil result<1.2 mg/dL should be reported when the measured I-index is<1.2. An I-index >1.2 will be suggestive of hyperbilirubinemia and TBil quantification will be performed.

This cut-off value (1.2) was the one that showed the highest sensitivity (100 %) and specificity (87 %). A 1.2 cut-off value ensures 100 % sensitivity, thereby resulting in a 0 % false negative rate. This way, no patient will be mistakenly considered healthy and no patient with hyperbilirubinemia will be considered healthy.

The false positive rate will be higher with the 1.2 cut-off value, resulting in a 87 % specificity. Otherwise said, a higher proportion of patients will have results consistent with hyperbilirubinemia and TBil testing will be required to confirm or exclude the presence of hyperbilirubinemia [4].

The AUC demonstrates that the I-index is an effective method for separating patients with hyperbilirubinemia from those without this condition.

From the 3,194 determinations performed in January, 2,536 (79.39 %) were unnecessary. The cost of a TBil test is 0.05 Euros.

The high correlation observed between the I-index and TBil supports its use as a reliable and effective tool for the preliminary screening of hyperbilirubinemia. This will allow to optimize resources, reduce unnecessary costs and improve diagnostic performance while guaranteeing patient safety [5], 6].
